# Establishment and evaluation of an early prediction model of hepatorenal syndrome in patients with decompensated hepatitis B cirrhosis

**DOI:** 10.1186/s12876-022-02618-x

**Published:** 2023-01-02

**Authors:** Shouhao Wang, Zhewen Zhou, Chengan Xu, Hanzhu Chen, Wenya Ren, Xingdi Yang, Qiaoqiao Yin, Wei Zheng, Hongying Pan

**Affiliations:** 1 Department of Infectious Diseases, Zhejiang Provincial People’s Hospital, Qingdao University, 310014 Hangzhou, Zhejiang China; 2 Center for General Practice Medicine, Department of Infectious Diseases, Zhejiang Provincial People’s Hospital (Affiliated People’s Hospital, Hangzhou Medical College), No. 158 Shangtang Road, Hangzhou, 310014 Zhejiang China

**Keywords:** Hepatorenal syndrome, Hepatitis B virus, Cirrhosis, Predictors, Retrospective study, Model

## Abstract

**Background and aim:**

In China, hepatorenal syndrome is a serious complication in the decompensated stage of hepatitis B cirrhosis, which requires early clinical intervention, so the early diagnosis of hepatorenal syndrome is crucial. This study establishes a new predictive model based on serum biomarkers for the early diagnosis of hepatorenal syndrome.

**Methods:**

Patients with decompensated hepatitis B cirrhosis who met the inclusion and exclusion criteria were retrospectively enrolled. Patients were randomly assigned to the training dataset and validation dataset at a 7:3 ratio. Univariate and multivariate logistic regression analyses were used to screen the risk factors for hepatorenal syndrome. The identified risk factors were used to establish and verify a model.

**Results:**

This study included 255 patients with decompensated hepatitis B cirrhosis, including 184 in the training group and 71 in the validation group. The multivariate logistic regression model was established in the training group and verified in the validation group. Logistic regression showed that hemoglobin (OR 0.938, 95% CI 0.908–0.969), total bilirubin (OR 1.014, 95% CI 1.008–1.021) and creatinine (OR 1.079, 95% CI 1.043–1.117) were independent risk factors for hepatorenal syndrome (P < 0.05). These were used to establish the model. In the training group and the validation group, the area under the ROC curve of the nomogram for the diagnosis of hepatorenal syndrome was 0.968 and 0.980, respectively.

**Conclusion:**

The three serum biomarkers, including hemoglobin, total bilirubin and creatinine, can be used as independent early predictors of hepatorenal syndrome in patients with decompensated hepatitis B cirrhosis.

## Introduction

Hepatorenal syndrome (HRS) is a form of renal insufficiency and is one of the most common severe complications in the late stage of decompensated cirrhosis; it presents as decreased renal blood flow and a decreased glomerular filtration rate (GFR), without significant changes in renal histology [1]. In the past, HRS was considered to be a functional kidney injury, but with the development of pathophysiology, it is now believed that as a functional kidney injury, HRS will also progress to a structural kidney injury. In 2015, the International Ascites Club (IAC) proposed a new definition of acute kidney injury with cirrhosis in terms of the progression of HRS [2], and in 2019, the IAC proposed a new perspective based on the results of relevant clinical studies in recent years [3]. HRS is called hepatorenal syndrome-nonacute kidney injury (HRS-NAKI). Hepatorenal syndrome-acute kidney disease (HRS-AKD) and hepatorenal syndrome-chronic kidney disease (HRS-CKD) are subtypes of HRS-NAKI.

The pathogenesis of HRS is complex and involves many factors. First, portal hypertension dilates visceral blood vessels, leading to renal vasoconstriction, which leads to a decrease in the GFR [4]. Second, the increase in circulating inflammatory mediators [5], bacterial infection [5], cardiac dysfunction [6], and impaired renal tubule function [7] are also important mechanisms of HRS. HRS mostly manifests as acute renal failure in patients with severe liver disease such as cirrhosis, and it is also the main cause of death in advanced cirrhosis patients [8, 9]. The median survival time is 2 weeks to 2 months [10, 11]. Treatment of hepatorenal syndrome should be started as soon as possible after diagnosis. Liver transplantation is the best treatment for hepatorenal syndrome [12]. Drug therapy and renal replacement therapy are usually used as transitional treatments before liver transplantation. Among them, albumin combined with terisopressin is the current preferred drug treatment for hepatorenal syndrome [3, 13, 14]. At present, research on HRS mainly focuses on diagnosis and treatment, and studies on the prediction of HRS are limited, especially the prediction model. At the same time, China is a country with a high prevalence of hepatitis B [15], long-term HBV infection will damage the liver, resulting in a higher incidence rate of Compensated cirrhosis with hepatitis B, which further leads to a higher incidence of HRS in patients with compensated hepatitis B. Therefore, it is important to develop a predictive model for HRS in decompensated hepatitis B cirrhosis and timely intervention.

In this study, we aimed to construct a new model for the early prediction of HRS occurrence by retrospectively studying patients with decompensated hepatitis B cirrhosis from July 2018 to June 2021.

## Materials and methods

### Patients

This was a single-center, retrospective study. This study recruited patients with decompensated hepatitis B cirrhosis who were hospitalized in Zhejiang Provincial People’s Hospital (Hangzhou, China) between July 2018 and June 2021. Inclusion criteria: (1) patients with Decompensated cirrhosis with or without HRS according to the diagnostic criteria; (2) 18–90 years of age; and (3) Compliance with HRS diagnostic criteria. Exclusion criteria: (1) Patients infected with hepatitis A, C, D, or E virus; (2) Infection with human immunodeficiency virus (HIV); (3) Non-HBV with autoimmune hepatitis (AIH), primary biliary cholangitis (PBC), primary sclerosing cholangitis (PSC), hereditary metabolic liver disease, drug-induced liver injury (DILI) or alcoholic liver disease; (5) Hepatocellular carcinoma; or (6) Incomplete clinical data. The study was approved by the Ethics Committee of Zhejiang Provincial People’s Hospital and conformed to the ethical guidelines of the Helsinki Declaration. Researchers only analyzed anonymous data, so they gave up informed consent.

## Laboratory tests, clinical diagnosis and definitions

The baseline variables of patients, including age, sex, and clinical data, were collected. All included patients were subject to routine laboratory tests at admission, including routine blood tests, liver function, renal function, electrolytes, blood lipids, coagulation function, the use of diuretics, esophageal-gastro varices bleeding (EGVB) and bacterial infection. The routine blood examination indexes were white blood cells, neutrophil/lymphocyte ratio and platelets; the liver function indexes were albumin, globulin, alanine aminotransferase, aspartate aminotransferase, and total bilirubin; the renal function indexes were urea and creatinine; the electrolyte index was serum sodium concentration; and the coagulation function indexes were prothrombin time, international standardized ratio and activated partial thromboplastin time. The diagnosis of hepatitis B cirrhosis meet the following criteria (1) and (2) or (1) and (3) [16, 17]: (1) currently HBsAg positive, or HBsAg negative, anti-HBc positive, with a clear history of chronic HBV infection (previous HBsAg positive > 6 months), and excluding other causes; (2) patients whose liver biopsy pathology confirmed liver cirrhosis; and (3) two or more of the following five items were met, except for noncirrhotic portal hypertension: (1) imaging examination showed signs of cirrhosis and/or portal hypertension; (2) endoscopic examination showed esophageal and gastric varices; (3) liver hardness value was consistent with liver cirrhosis; (4) serum biochemical examination showed a decrease in albumin level; and (5) blood routine examination showed platelet count < 100 × 10^9^/L. Decompensated liver cirrhosis: Patients with liver cirrhosis are diagnosed with decompensated liver cirrhosis if they have serious complications such as ascites, gastroesophageal variceal bleeding or hepatic encephalopathy [18]. Its liver function mostly belongs to Child‒Pugh B or C grade. HRS diagnostic criteria: (1) Cirrhosis with ascites; (2) Comply with the diagnostic criteria of liver cirrhosis acute kidney injury (AKI). (3) 48 h of continuous discontinuation of diuretics and albumin (1 g kg^−1^ d^−1^) volume expansion therapy was ineffective; (4) No shock; (5) Not taking drugs that can damage the kidney (such as nonsteroidal anti-inflammatory drugs, aminoglycoside antibiotics, iodine contrast agents, etc.) (6) There were no obvious clinical signs of structural renal injury, namely, no proteinuria, no microhaematuria, and no abnormalities in renal imaging, but renal tubule and renal interstitial lesions were not completely excluded. Among them, protein excretion in urine > 500 mg/d, microscopic examination of micro hematuria finger urine sediment per high vision red blood cells < 50 [3].

### Statistical analysis

SPSS software (version 26.0, SPSS Inc., IBM, Chicago, IL, USA) was used for statistical analysis of the data. Quantitative variables are expressed as the mean ± standard deviation (SD) or median (25th percentile; 75th percentile) and were compared by independent t test or a nonparametric test (Mann–Whitney). Categorical variables are expressed as numbers (percentages) and were compared by the chi-square test. P < 0.05 was considered statistically significant. The variables that met the criteria in univariate analysis were introduced into multivariate stepwise logistic regression to construct a prediction model in the training set. Both univariate analysis and multivariate analysis showed that P < 0.05 was significantly different. The accuracy of the model for the diagnosis of HRS was evaluated by the area under the ROC curve (AUC). Welch-corrected t-test was used to analyze the model values of the HRS group and the non-HRS group in the training set, the validation set and the whole population. P < 0.05 indicated that the difference was statistically significant.

## Results

### General characteristics

**Fig. 1:**
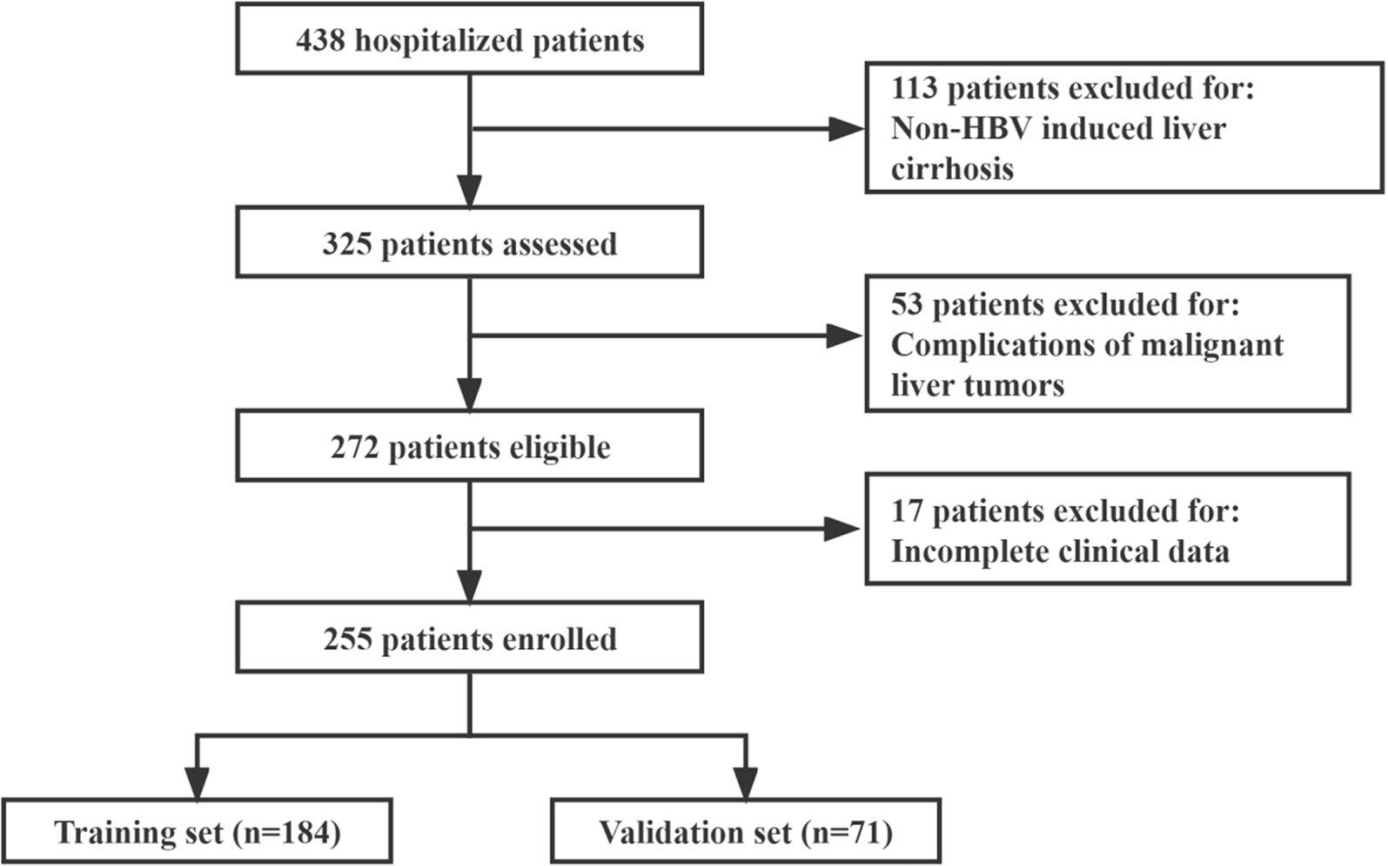
Flow diagram depicting the participant selection process

From July 2018 to June 2021, 438 patients with decompensated cirrhosis, including 325 patients with hepatitis B, were diagnosed in the Department of Infection, Zhejiang Provincial People’s Hospital. Fifty-three patients with malignant liver tumors were excluded from our study. Seventeen patients were excluded due to incomplete clinical data, which resulted in 255 patients being enrolled (Fig. 1). Among all patients, 46 patients had hepatitis B cirrhosis complicated with HRS, and 209 patients did not have HRS. The baseline characteristics are shown in Table 1. The average time from admission to HRS diagnosis in all patients with hepatitis B cirrhosis complicated with HRS was 4.4 days; the longest time was 30 days, and the shortest time was 2 days. A total of 255 patients were randomly divided into a training group and a validation group at a ratio of 7:3. The training group had 184 patients with decompensated hepatitis B cirrhosis, of whom 35 had HRS. There were 71 patients with decompensated hepatitis B cirrhosis in the validation group, including 11 patients with HRS. The baseline characteristics of all enrolled patients are listed in Table 2. There was no significant difference in the parameters between the training set and the validation set (P > 0.05).

**Table 1:** Characteristics of patients in the HRS and non-HRS sets at baseline

Variable	HRS set (n = 46)	non-HRS set (n = 209)	P value
Age (year)	54 (51, 62)	54 (49, 60)	0.788
Male sex (%)	30 (65.2%)	155 (74.2%)	0.218
WBC (10^9^/L)	5.33 (3.75, 7.24)	3.18 (2.16, 4.98)	< 0.001
NLR	5.25 (2.60, 7.92)	2.27 (1.52, 3.50)	< 0.001
Hb (g/L)	88.2 ± 25.6	106.6 ± 23.5	< 0.001
PLT (10^9^/L)	67.0 (46.5, 87.5)	63.0 (42.0, 96.5)	0.614
ALB (g/L)	29.1 (26.3, 31.9)	27.6 (24.3, 32.5)	0.396
GLB (g/L)	30.1 (26.3, 34.2)	30.7 (27.2, 36.2)	0.214
ALT (U/L)	32 (21, 60)	26 (20, 40)	0.121
AST (U/L)	62 (34, 99)	39 (30, 58)	0.025
GGT (U/L)	53 (25, 93)	28 (19, 51)	0.002
ALP(U/L)	150.00 (113.0, 172.5)	112 (82, 146)	< 0.001
TB (µmol/L)	205.0 (54.4, 383.6)	30.8 (21.2, 50.0)	< 0.001
BUN (mmol/L)	7.46 (5.69, 10.87)	4.72 (3.85, 6.01)	< 0.001
Cr (µmol/L)	108.4 (84.0, 128.5)	70.2 (62.5, 82.3)	< 0.001
Na^+^ (mmol/L)	137.75 (133.95, 140.38)	139.90 (137.40, 141.60)	0.001
PT (s)	19.05 (16.05, 25.10)	15.60 (13.85, 17.05)	< 0.001
INR	1.84 (1.51, 2.30)	1.43 (1.26, 1.59)	< 0.001
APTT (s)	44.30 (36.88, 55.55)	34.10 (30.25, 39.45)	< 0.001
Use of diuretics (%)	33 (71.7%)	132 (63.2%)	0.270
EGVB (%)	8 (17.4%)	33 (15.8%)	0.789
Bacterial infection (%)	33 (71.7%)	75 (26.1%)	< 0.001

Quantitative variables are expressed as median (25th percentile; 75th percentile)

Categorical variables are expressed as numbers (percentages)

*WBC* white blood cell count, *NLR* neutrophil to lymphocyte ratio, *Hb* hemoglobin, *PLT* platelet count, *ALB* albumin, *GLB* globulin, *ALT* alanine transaminase, *AST* aspartate transaminase, *TB* total bilirubin, *BUN* blood urea nitrogen, *Cr* creatinine, *Na*^+^ serum sodium ion concentration, *PT* prothrombin time, *INR* international normalized ratio, *APTT* activated partial thromboplastin time, *EGVB* esophageal-gastro varices bleeding

**Table 2:** Characteristics of patients in the training and validation sets at baseline

Variable	Training set (n = 184)	Validation set (n = 71)	P value
Age (years)	53 (49, 58)	54 (48, 62)	0.432
Male sex (%)	130 (70.7%)	55 (77.5%)	0.275
WBC (10^9^/L)	3.64 (2.22, 5.50)	3.31 (2.37, 5.03)	0.415
NLR	2.47 (1.67, 4.54)	2.50 (1.50, 4.50)	0.690
Hb (g/L)	102.85 ± 24.573	104.39 ± 245.724	0.657
PLT (10^9^/L)	65 (43, 88)	60 (42, 111)	0.740
ALB (g/L)	28.15 (24.50, 32.58)	27.9 (25, 31.9)	0.836
GLB (g/L)	30.35 (27.05, 35.25)	31.10 (26.70, 36.40)	0.509
ALT (U/L)	26 (20, 42)	29.00 (20.00, 44.00)	0.556
AST (U/L)	39 (29, 63)	42.00 (31.00, 74.00)	0.352
TB (µmol/L)	35.95 (21.525, 77.975)	34.3 (21.8, 65.90)	0.709
BUN (mmol/L)	5.02 (3.93, 7.19)	4.83 (3.84, 6.06)	0.187
Cr (µmol/L)	74.45 (63.4, 91.425)	74.10 (65.10, 84.50)	0.602
Na^+^ (mmol/L)	139.70 (136.8, 141.50)	139.70 (137.90, 141.00)	0.648
PT(s)	15.95 (14.025, 17.8)	16.10 (14.00, 18.10)	0.796
INR	1.48 (1.2725, 1.68)	1.47 (1.28, 1.67)	0.928
APTT (s)	36.25 (30.625, 42.475)	34.80 (31.10, 41.00)	0.655
Use of diuretics (%)	115 (62.5%)	50 (70.4%)	0.235
EGVB (%)	31 (16.8%)	10 (14.1%)	0.590
Bacterial infection (%)	74 (40.4%)	34 (47.9%)	0.281

Quantitative variables are expressed as the mean ± standard deviation

Categorical variables are expressed as numbers (percentages)

## Predictors of HRS and formulation of the model

The results of univariate logistic regression analysis with HRS as the dependent variable showed that WBC, NLR, Hb, GLB, TB, BUN, serum creatinine, Na^+^, PT, APTT and infection might be influencing factors of HRS in patients with decompensated hepatitis B cirrhosis (P < 0.05) in the training set (Table 3). The independent variables with statistically significant differences (P < 0.05) in univariate logistic regression were included in the multivariate logistic regression (stepwise forward/maximum likelihood ratio method). The results suggested that Hb, TB and serum creatinine were independent risk factors for HRS in patients with decompensated hepatitis B cirrhosis (P < 0.05). The corresponding predictive model was constructed (Table 4). The model formula was as follows (hemoglobin is in g/L, total bilirubin is in µmol/L, and creatinine is in µmol/L):

$$\text{Model}=-0.064\times\text{Hb}+0.014\times\text{TB}+0.076\times\text{creatinine}-3.802.$$

**Fig. 2:**
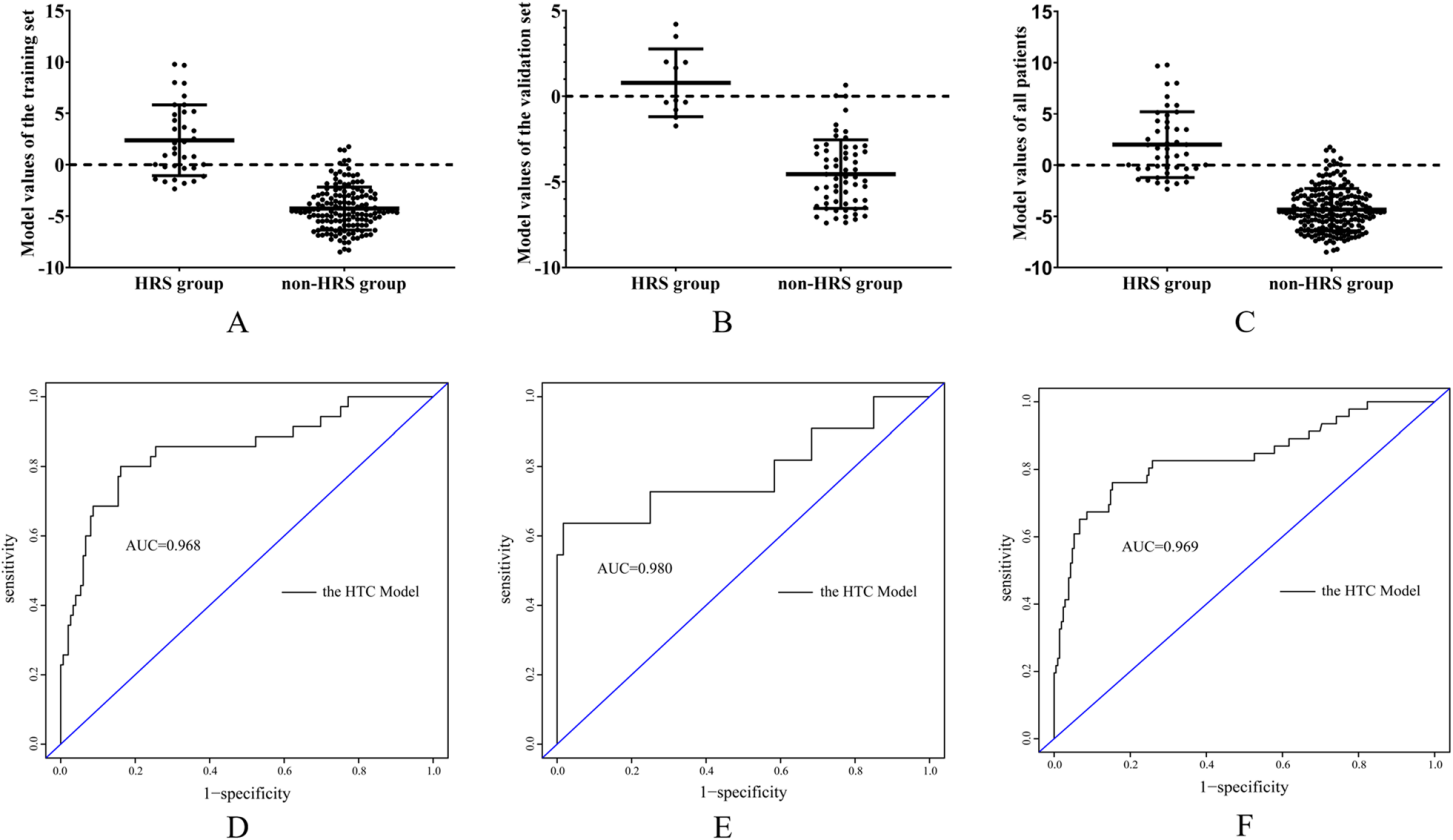
ROC curves of model values and early diagnosis models in patients with decompensated hepatitis B cirrhosis in the HRS group and the non-HRS group. **A** In the training set, the model value of the HRS group was significantly higher than that of the non-HRS group (t = 10.350, P < 0.0001). **B** In the validation set, the model value of the HRS group was significantly higher than that of the non-HRS group (t = 8.213, P < 0.0001). **C** In all patients, the model value in the HRS group was significantly higher than that in the non-HRS group (t = 11.830, P < 0.0001). **D** The ROC curve [area under the ROC curve (AUC)] of the model in the training set was 0.968. **E** ROC curve of the model in the validation set (AUC 0.980). **F** ROC curve of the model in all patients (AUC 0.969)

In the training group (t = 10.350, P < 0.0001), the validation group (t = 8.213, P < 0.0001) and the total group (t = 11.830, P < 0.0001), the model score of the HRS group was significantly higher than that of the non-HRS group (Fig. 2A–C).

**Table 3:** Univariate logistic regression analysis of influencing factors of hepatorenal syndrome in patients with decompensated hepatitis B cirrhosis in the training set

Variable	Coefficient	P value	OR	95% CI
Age (year)	0.020	0.296	1.020	0.983–1.059
Male sex (%)	0.285	0.477	1.329	0.607–2.911
WBC (10^9^/L)	0.195	< 0.001	1.215	1.097–1.346
NLR	0.142	0.002	1.153	1.053–1.263
Hb (g/L)	− 0.034	< 0.001	0.967	0.950–0.984
PLT (10^9^/L)	0.002	0.517	1.002	0.996–1.007
ALB (g/L)	0.013	0.654	1.013	0.956–1.074
GLB (g/L)	− 0.081	0.017	0.922	0.862–0.985
ALT (U/L)	0.002	0.135	1.002	0.999–1.006
AST (U/L)	0.005	0.056	1.005	1.000–1.009
TB (µmol/L)	0.011	< 0.001	1.011	1.007–1.015
BUN (mmol/L)	0.250	< 0.001	1.284	1.157–1.425
creatinine (µmol/L)	0.074	< 0.001	1.077	1.053–1.101
Na^+^ (mmol/L)	− 0.169	0.001	0.845	0.764–0.934
PT (s)	0.288	< 0.001	1.334	1.191–1.483
INR	0.164	0.220	1.178	0.907–1.530
APTT (s)	0.096	< 0.001	1.101	1.059–1.144
Use of diuretics (%)	0.494	0.228	1.639	0.734–3.660
EGVB	0.264	0.581	1.302	0.510–3.321
Bacterial infection	1.453	< 0.001	4.276	1.939–9.430

Categorical variables are expressed as numbers (percentages)

**Table 4:** Multivariate logistic regression analysis of independent risk factors for hepatorenal syndrome in patients with decompensated hepatitis B cirrhosis in the training set

Variable	Coefficient	P	OR	95% CI
Hb	− 0.064	< 0.001	0.938	0.908–0.969
TB	0.014	< 0.001	1.014	1.008–1.021
Cr	0.076	< 0.001	1.079	1.043–1.117
Constant	− 3.802	0.039	0.022	0.000–0.000

Figure 2D shows the ROC curve of the model for the early prediction of HRS in decompensated hepatitis B cirrhosis patients, with an AUC of 0.968 [standard error (SE) 0.011, 95% confidence interval (CI) 0.946–0.989]. The ROC curve of the model for the validation set is shown in Fig. 2E, and its AUC was 0.980 [standard error (SE) 0.014, 95% 0.951–1.000]. The ROC curve of the model for all patients is shown in Fig. 2F, with an AUC of 0.969 (SE 0.009, 95% CI 0.951–0.987).

Based on ROC analysis, the best cutoff value of the model was calculated. When the high cutoff value (> 0.146) is selected to determine which patients have HRS, symptomatic treatment is needed. A lower cutoff value (< 0.146) was used to determine whether patients without HRS could be followed up regularly. The sensitivity, specificity, positive predictive value and negative predictive value of the model were calculated (Table 5). In the training group, when the critical value of the model was 0.146, 22 patients (62.9%) were recommended to start symptomatic treatment, and 13 patients were regularly followed up.

**Table 5:** The diagnostic accuracy of the model for the early prediction of HRS

	AUR	*P* value	Cutoff values	95% CI	Se%	Sp%
Training set	0.968	P < 0.001	0.146	0.949–0.992	97.1	87.2
Validation set	0.980	P < 0.001	0.157	0.954-1.000	100	91.7
All patients	0.969	P < 0.001	0.150	0.951–0.987	97.8	88.5

*Se* sensitivity, *Sp* specificity

The model performed well on the training set and verification set, with AUCs of 0.968 and 0.980, respectively. A total of 46 patients with decompensated hepatitis B cirrhosis were complicated with HRS in the entire population, of whom 30 (65.2%) could be treated early according to the critical value.

**Fig. 3:**
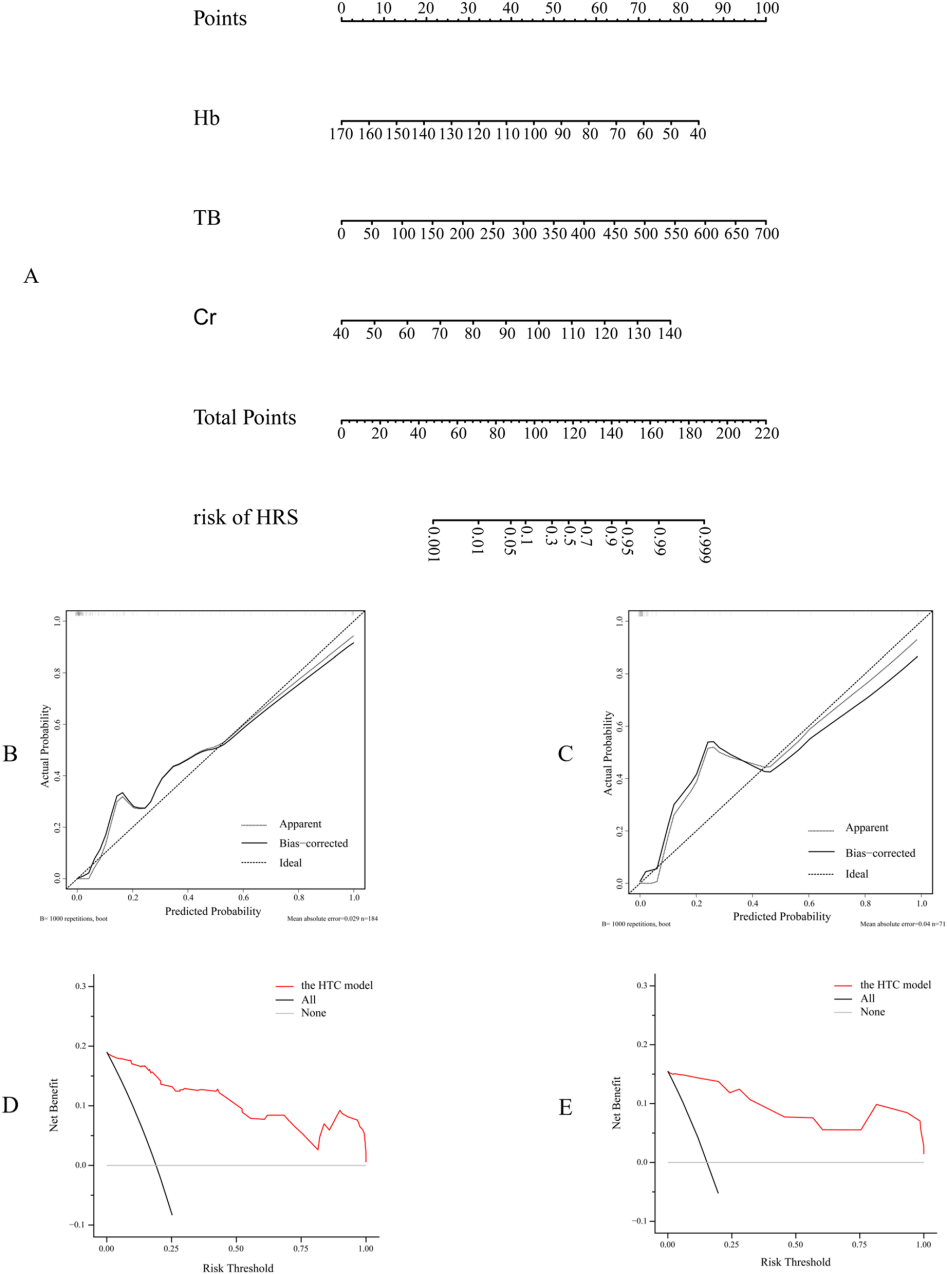
Nomo map based on the training group, calibration curve and decision curve based on the validation set. ** A** Displaying the model with visual charts is more suitable for clinical application. ** B**, **C** Validation of the nomogram prediction accuracy by building calibration curves using validation sets. **D**, **E** The decision curve of the validation set shows that the prediction model has obvious net benefits, indicating that the model has high clinical application value

To facilitate clinical application, this study provided a visualization of the prediction model and established the nomogram (Fig. 3A), which can more intuitively express the relationship between the three variables in the prediction model. The calibration curves of the training and validation groups are shown in Fig. 3B, C, and the predicted risk and the actual risk are in good agreement. Additionally, to evaluate the clinical effectiveness of the model, this study drew a decision curve (Fig. 3D, E). The analysis showed that the scoring prediction model had obvious net benefits, suggesting that the prediction model had high clinical application value and met the actual needs of clinical decision-making. Since the AUC of the model in the training group was relatively high and the calibration curve and the decision curve were good, the collinearity of the prediction indexes of the training group was judged. The variance inflation factors (VIFs) of the predictive factors (Hb, TB and serum creatinine) were all less than 5, indicating no collinearity.

## Discussion

The prevalence of hepatitis B virus is a major public health problem not only in China but also worldwide. For patients with decompensated cirrhosis, hepatorenal syndrome is one of the serious complications. Therefore, early prediction and diagnosis of HRS can reduce the incidence [19] and severity [20] of HRS by allowing for early control and prevention of infection, correction of anemia, avoidance of nephrotoxic drugs, and maintenance of blood volume balance.

HRS is a serious complication of renal function damage without obvious organic disease in the kidney that may occur in patients with advanced severe liver disease, and the incidence rate of HRS in patients with cirrhosis is about 19.2%. In this study, we tried to establish a diagnostic model to predict HRS by retrospective analysis of clinical data of HRS patients. It should be pointed out that the laboratory indicators included in this study are all indicators that must be tested on admission, so some unconventional indicators related to renal injury are not included, such as Urine electronics and urine β 2-MG concentrations. Janicko et al. included urine sodium in a study on predictors of alcohol-related cirrhosis with hepatorenal syndrome, but the difference was not statistically significant [21]. At the same time, urinary sodium was not used as a routine test indicator at admission, so we did not include urinary electrolytes in the study of prediction indicators.

In addition, some biomarkers whose predictive value for HRS has not been clearly defined are also excluded from the model established in this study, such as serum cystatin C (CysC) [22, 23], urine neutrophil gelatinase-associated lipocalin (NGAL) [24], serum interleukin-18 (IL-18) [25, 26], serum N-acetyl-β-D glucosaminidase (NAG) [23], urinary kidney injury molecule-1 (KIM-1) [27] and liver-type fatty acid binding protein (LFABP) [28], have been proposed for the detection of HRS [29]. Although these markers have been shown to increase in HRS in patients with cirrhosis [27, 30, 31], the predictive value of these markers for HRS has not been well studied in decompensated cirrhosis patients with normal serum creatinine levels [19]. Therefore, we performed a retrospective study in patients with decompensated hepatitis B cirrhosis with normal serum creatinine levels to study the predictive model for the early prediction of HRS development.

**Fig. 4:**
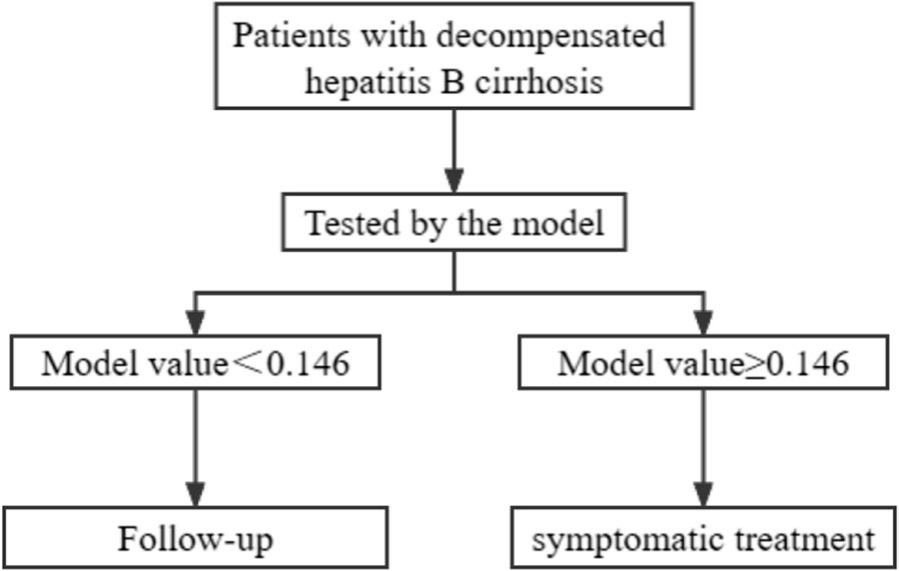
Application of our model for the early prediction of decompensated hepatitis B cirrhosis patients with HRS symptomatic treatment algorithm

In this study, we constructed a noninvasive model of early HRS in patients with decompensated hepatitis B cirrhosis and a specific algorithm (Fig. 4). The model was constructed with three independent predictors: Hb, TB and scr. The method successfully predicted the diagnosis of 22 training set patients (63%), 5 validation set patients (45%) and 27 full-cohort patients (59%). Therefore, it can predict concurrent HRS in advance, allowing for earlier symptomatic treatment and improving prognosis. Alessandria C et al. also established a model to predict HRS, that is, the diagnostic value of the model of end-stage liver disease (MELD) score Compared with the prediction model of Alessandria C et al., the AUC value of the prediction model established in this study is higher (0.968 vs. 0.896), but the prediction model in this study relies on serum creatinine, while the model of Alessandria C et al. does not rely on serum creatinine.

The following might explain the clinical significance and predictive value of Hb, TB and Cr for the early prediction of HRS. First, anemia is common in patients with advanced liver disease. When the MELD score increases, hemoglobin levels decrease [29]. Anemia can reduce the oxygen supply of the kidney and may lead to microcirculation renal hypoxia or injury, which can cause early renal tubular injury [32, 33], and renal ischemia is the most common cause of HRS. Studies have found that anemia can aggravate HRS in patients with liver cirrhosis [34]. In patients with end-stage renal disease, there was a strong positive correlation between anemia and the risk of adverse prognosis [35]. In this study, we found that Hb was one of the influencing factors for the early prediction of concurrent HRS, which was similar to the above research results.

Second, total bilirubin is divided into direct bilirubin and indirect bilirubin. Most human bilirubin comes from hemoglobin released from aging red blood cells. Indirect bilirubin is transported to the liver through blood and generates direct bilirubin through the action of hepatocytes. The occurrence of liver diseases will affect this process. Serum total bilirubin is an index reflecting the reserve function of the liver. The worse the reserve function of the liver, the more likely patients are to have HRS. Therefore, patients with liver cirrhosis who have obvious liver function damage should be aware of the potential occurrence of HRS. Studies have found that serum total bilirubin concentration may be a new risk factor for chronic kidney disease [36], and serum bilirubin concentration is positively correlated with renal dysfunction [37, 38]. Therefore, we assessed the association between TB and HRS risk and found that TB was one of the factors that could predict concurrent HRS early.

Finally, most guidelines define HRS based on serum creatinine. Adding serum creatinine to the MELD score can reflect the prognostic impact of renal function, predict the likelihood of death within 3 months and rank American patients for organ transplants [39, 40]. In this study, it was found that serum creatinine was one of the influencing factors for the early prediction of concurrent HRS. These results are similar to previous research results [41].

Our prediction model has unique characteristics. It uses only conventional serum markers, which are inexpensive and easy to obtain. Therefore, it would be useful in most medical institutions. At the same time, the diagnostic efficiency (AUC = 0.968) was better than that of the MELD score (AUC = 0.896) [10]. Although the MELD score can effectively evaluate the severity of advanced liver diseases and liver graft allocation [42], it has not been used for the early prediction of HRS.

This study also has several limitations. First, the sample size is relatively small. Before its clinical application, large cohort studies should be conducted. Second, the model was built in a training group, and another group in the same center was randomly selected for validation. Third, serum-specific test indicators can improve the effectiveness of the model. Nonetheless, we believe that external validation of multicenter studies can enhance the effectiveness of the model. However, our main goal is to build a noninvasive model for use in most medical facilities. Many hospitals have no access to data on special test indexes in serum, which limits clinical application.

## Conclusion

This study showed that Hb, TB and Cr were independent risk factors for hepatorenal syndrome in patients with decompensated hepatitis B cirrhosis. A simple, rapid, personalized and accurate diagnostic method for HRS is provided by the line chart established by serological indicators and is worthy of application in clinical trials. However, despite the innovativeness of this study, we still need to conduct multicenter and large-sample studies to confirm our findings and develop more effective methods for the early diagnosis of hepatorenal syndrome using line maps.

## Data Availability

The datasets used and/or analysed during the current study are available from the corresponding author on reasonable request.

## Abbreviations

OR: Odds ratio
95% CI: 95% Confidence interval
ROC: Receiver operator curve
AUC: Area under the ROC curve
HRS: Hepatorenal syndrome
GFR: Glomerular filtration rate
IAC: International Ascites Club
HBV: Hepatitis B virus
SE: Standard error
MELD: Model of end-stage liver disease
WBC: White blood cell count
NLR: Neutrophil to lymphocyte ratio
Hb: Hemoglobin
PLT: Platelet count
ALB: Albumin
GLB: Globulin
ALT: Alanine transaminase
AST: Aspartate transaminase
TB: Total bilirubin
BUN: Blood urea nitrogen
Cr: Creatinine
Na+: Serum sodium ion concentration
PT: Prothrombin time
INR: International normalized ratio
APTT: Activated partial thromboplastin time
EGVB: Esophageal-gastro varices bleeding
